# 25 Years of the International Bipolar Collaborative Network (BCN)

**DOI:** 10.1186/s40345-020-00218-w

**Published:** 2021-04-02

**Authors:** Robert M. Post, Lori L. Altshuler, Ralph Kupka, Susan L. McElroy, Mark A. Frye, Heinz Grunze, Trisha Suppes, Paul E. Keck, Willem A. Nolen

**Affiliations:** 1 Bipolar Collaborative Network, 5415 W Cedar Lane, Ste 201-B, Bethesda, 20814 MD USA; 2 Department of Psychiatry and Behavioral Sciences, George Washington University, Washington, D.C. USA; 3 Department of Psychiatry and Biobehavioral Sciences, David Geffen School of Medicine, University of California, Los Angeles, CA USA; 4 Department of Psychiatry, VA Greater Los Angeles Healthcare System, West Los Angeles Healthcare Center, Los Angeles, CA USA; 5 Department of Psychiatry, Amsterdam UMC, Vrije Universiteit, Amsterdam, The Netherlands; 6 Lindner Center of HOPE, Mason, OH USA; 7 Biological Psychiatry Program, University of Cincinnati Medical College, Cincinnati, OH USA; 8 Department of Psychiatry& Psychology, Mayo Clinic, Rochester, MN USA; 9 Psychiatrie Schwäbisch Hall GmbH & Paracelsus Medical University, Nuremberg, Germany; 10 Department of Psychiatry and Behavioral Sciences, Stanford University School of Medicine, Palo Alto, CA USA; 11 V.A. Palo Alto Health Care System, Palo Alto, CA USA; 12 Department of Psychiatry, University Medical Center Groningen, University of Groningen, Groningen, The Netherlands

**Keywords:** Bipolar disorder, Comorbidities, Depression, Epigenetics, Mania, Obesity, Offspring, Background

## Abstract

**Background:**

The Stanley Foundation Bipolar Treatment Outcome Network (SFBN) recruited more than 900 outpatients from 1995 to 2002 from 4 sites in the United States (US) and 3 in the Netherlands and Germany (abbreviated as Europe). When funding was discontinued, the international group of investigators continued to work together as the Bipolar Collaborative Network (BCN), publishing so far 87 peer-reviewed manuscripts. On the 25th year anniversary of its founding, publication of a brief summary of some of the major findings appeared appropriate. Important insights into the course and treatment of adult outpatients with bipolar disorder were revealed and some methodological issues and lessons learned will be discussed.

**Results:**

The illness is recurrent and pernicious and difficult to bring to a long-term remission. Virtually all aspects of the illness were more prevalent in the US compared to Europe. This included vastly more patients with early onset illness and those with more psychosocial adversity in childhood; more genetic vulnerability; more anxiety and substance abuse comorbidity; more episodes and rapid cycling; and more treatment non-responsiveness.

**Conclusions:**

The findings provide a road map for a new round of much needed clinical treatment research studies. They also emphasize the need for the formation of a new network focusing on child and youth onset of mood disorders with a goal to achieve early precision diagnostics for intervention and prevention in attempting to make the course of bipolar illness more benign.

**Supplementary Information:**

The online version contains supplementary material available at 10.1186/s40345-020-00218-w.

Ted and Vada Stanley funded our Stanley Foundation Bipolar Network (SFBN, in short Network) from 1995 to 2002 through a grant managed by E. Fuller Torrey. We recruited 935 outpatients with bipolar disorders who were rated extensively including with daily ratings by clinicians on the NIMH-Life Chart Method (LCM). The numbers of patients with continuous prospective LCM-ratings was: at least 1 year of follow-up = n = 539; 2 years = n = 315; 3 years n = 207; 4 years n = 130; 5 years n = 84. Thus, the vast majority remained in the Network for more than 1 year and many participated in clinical trials intermittently while being engaged in ongoing naturalistic treatment.

When funding lapsed and recruitment into the Network ended in 2002, investigators from the US and Europe decided to stay in contact and continue the work of the Network, including data analysis and publication of the findings in what has since then been called the Bipolar Collaborative Network (BCN, also in brief Network)). A statistician and research assistant were funded for this purpose through an anonymous private donor arranged by Dr. Fred Goodwin.

The Network has been extremely productive with now 87 peer-reviewed publications (see Additional file 1) and its 25-year anniversary appears to be an appropriate time to summarize a few of its accomplishments and findings. This selective summary is intended to serve several purposes and to deliver some key messages.

The field for better understanding and development of clinical therapeutics of bipolar disorder has been vastly underfunded over the past 3 decades. The only other longitudinal network for its study was the NIMH-funded Systematic Treatment Enhancement Program for Bipolar Disorder (STEP-BD) from 1999 to 2005 which was also terminated prematurely for political and, to a lesser extent, financial reasons (Bowden et al. 2012). Our hope is that summarizing the findings of the Network will renew interest in forming future longitudinal networks designed to begin to address the many gaps in the understanding and treatment of bipolar disorder. Some of the methodological decisions and compromises made in the BCN are noted as they might be relevant to future studies.

Moreover, the findings of our Network have made it clear that bipolar disorder in the US (patients recruited from sites in Los Angeles, Dallas, Cincinnati, and Bethesda) is a much more pernicious disorder than it is in the other two countries involved in the Network (Utrecht, and surrounding areas of the Netherlands, and Freiburg and Munich, Germany; abbreviated here as “Europe”). This special untoward disadvantage needs to be systematically addressed with new research and public health endeavors.

One of the most striking findings in the Network is that two thirds of bipolar disorder in US patients began prior to age 19, while only one third had these onsets in childhood and adolescence in Europe (Post et al. 2017a). Similar to the findings in STEP-BD (Perlis et al. 2004), we found that patients with childhood onsets did much more poorly throughout adulthood. They had more episodes, more ultradian cycling days, more suicidality, more anxiety, more substance abuse and greater comorbidity generally (Leverich et al. 2007; Post et al. 2017a). At the same time the literature is sparse as to how childhood onset bipolar disorder is best treated so that children in the US too often have delayed or inadequate treatment. Treatment delay itself is an independent risk factor for a poor outcome (Post et al. 2010b). Further, in the absence of a mood stabilizer, the rates of antidepressant-induced mania in this patient population are higher than in adults, underscoring the negative impact of a clinical research void in this age group (Croarkin et al. 2017; Frye et al. 2015). These data are likely compounded by the fact that the presenting pole of bipolar disorder is often depression.

Therefore, another hoped for outcome of this summary would be the impetus for the formation of a longitudinal treatment outcome network for children and youth so that the knowledge gap in the literature could more rapidly be filled. The multicenter “Course and Outcome of Bipolar Youth (COBY)” study has given many insights in the course of childhood onset bipolar disorder and the potential role of lithium treatment (Birmaher et al. 2009; Hafeman et al. 2019) but now randomized (double-blind or open) and pragmatic clinical trials are needed in order to delineate what treatments (lithium and others) are most effective and best tolerated for use in prevention and in early treatment. Without such a sustained longitudinal trial network for those with early onset bipolar and related disorders, more generations will continue to suffer the avoidable consequences of an inadequate evidence-based treatment portfolio.

## Methods

### Network design and patient population

Outpatients with bipolar disorder (75% bipolar I), average age about 40, gave informed consent for Network participation and separate consents for any embedded systematic clinical trial. They were seen weekly to monthly depending on the severity of symptoms. At each visit, they were rated on cross sectional measures of depression (IDS-C) and mania (YMRS) and functioning on the Global Assessment Scale (GAS), and self- rated daily using the LCM. At entry into the Network they also filled out a detailed patient questionnaire about demographics, relevant aspects of personal history, family history, and prior course of illness. All patients received state-of-the-art treatment for BD and comorbid conditions during their participation in the naturalistic follow-up study.

## Results

### Naturalistic follow-up study

Patients in the Network showed considerable morbidity, with number of prior episodes being one of the biggest factors associated with a poor outcome assessed prospectively (Post et al. 2010a). Moreover, compared to patients in Europe, those from the US had a higher incidence of abuse in childhood, an early age of onset of bipolar disorder, more anxiety disorder comorbidity, and more alcohol abuse and substance abuse comorbidity. The also had more frequently a history with 20 or more prior episodes, more rapid cycling, and more treatment non-response (for at least 6 months) during prospective naturalistic treatment (Table 1) (Post et al. 2014a). The detailed longitudinal course of the illness was documented as extremely pleiomorphic and characterized by every variation in cycle frequency that one could imagine.

**Table 1:** Comparison of BCN participants in the US and Europe

Clinical characteristics	United States (N = 676)	Europe (N = 292)
Early onset (< 19 y)	69.2%***	32.3%
Delay to first treatment for depression (age of onset < 19 y)	13.1 y*	8.4 y
Delay to first treatment for mania (age of onset < 19 y)	11.3y***	5.8y
Prospective nonresponders	51.7%***	31.1%
Life time history of		
Anxiety disorder	46.6%***	28.1%
Alcohol abuse	33.1%***	14.7%
Substance Abuse	38.3%***	17.8%
Rapid Cycling	74.1%***	41.5%
> 20 Episodes	59.0%***	23.3%
Hospitalizations	Fewer**	More

Burden of illness appears significantly higher in the US subjects (*p < 0.05; **p < 0.01; ***p =  < 0.001)

During treatment in the Network, we assessed three patterns of long- term responsiveness (Post et al. 2010a). We examined those who were well on admission and who then remained in essential remission for the next six months. This occurred in 96 of the 525 patients (18%) assessed longitudinally in the study. Of the 429 patients who were ill at Network entry, we divided them into good to excellent Responders for 6 months or more (45.5%), or Non-Responders who did not achieve a good response for a minimum of 6 months (54.5%). The Responders were treated with an average of 2.98 medications when they achieved criteria. Multiple medication revisions were required, and it took a mean duration of 18 months in the Network to become a Responder. Lithium and valproate were the drugs most often used in the Responders.

Non-Responders were treated unsuccessfully with many more drug trials in an attempt to achieve a good long-term outcome, but such a regimen was not found for these individuals. Non-Responders were ultimately exposed to more antidepressants and antipsychotics than the sustained responders. They tended to have a more adverse course of illness prior to Network entry (more episodes and rapid cycling) and were over-represented by patients who were from the US (Post et al. 2017a).

### Factors associated with the high incidence of early onset illness in the US

Among many other potential factors, two stood out as being associated with the high incidence of childhood onset bipolar disorder in the US. One was environmental and the other genetic. There was an added combined effect of a history of abuse in childhood and the family loading for psychiatric illness in patients’ parents and grandparents on age of onset of bipolar disorder. In those without either of these two vulnerability factors, the average of onset of bipolar disorder in the Network was 26 years of age. However, for those who showed both a high loading of family history of psychiatric illness and a high incidence of adversity in childhood, the age of onset of bipolar disorder in the Network tended to average 13 years of age or younger (Post et al. 2016b).

### Four generations of relatives of US patients were more ill than the Europeans

We examined the family history of depression, bipolar, suicide attempts, alcohol abuse, substance abuse, and “other” illnesses in multiple relatives of Network patients. There was a higher incidence of virtually all of these familial psychiatric difficulties in those from the US compared to the Europeans. This increased incidence of illness extended across four generations and included more illness in the US in: 1. Grandparents; 2. Parents; 3. Patients themselves and their Siblings and Spouses; and 4. The patients’ Offspring (Table 2) (Post et al. 2016a, 2017a).

**Table 2:** Comparison of the offspring of BCN participants in the US and Europe demonstrating a greater burden of illness in the US subjects

Offspring Dx	United States	Europe
Unipolar Depression	26.5%	8.9%
Bipolar Disorder	17.8%	3.8%
Suicide Attempt	6.0%	2.2%
Alcohol Abuse	7.2%	1.4%
Substance Abuse	12.0%	2.1%
Other	24.9%	5.1%
Any Illness	36.3%	13.3%

This burden of illness in the US could reflect two types of inherited vulnerability- that traditionally mediated by a parent with an illness and that when both parents had an affective disorder (bilineal vulnerability). Compared to the Europeans, patients from the US had three times more assortative mating and this same proclivity was also seen in the marriages of the patients’ parents (Post et al. 2020a).

### Psychosocial stress in childhood, at illness onset, and prior to the most recent episode

Compared to the Europeans, patients from the US experience more and more severe adversity in childhood, including verbal, physical, and sexual abuse. This adversity was associated with an earlier age of onset of bipolar disorder as well as a more adverse course of illness into adulthood. Most interestingly when we examined patients who only experienced verbal abuse (and did not have physical or sexual abuse), this isolated verbal abuse alone was still associated with an earlier age of onset and a more adverse course of illness (Post et al. 2015b).

In addition, those from the US experienced more interpersonal, economic, and medical stressors in the year prior to their first episode and then again in the year prior to the last episode they experienced prior to Network entry. Thus, it appeared that patients from the US had more stressors and accumulated more stressors over their course of their illness (Post et al. 2013a). The role of the differences in the national health care systems in the US compared to Europe likely contributed to the increased incidence of these health care, medical, and economic stressors. The availability of socialized healthcare, wage support and disability benefit provisions which are much more generous and easily accessible in Germany and the Netherlands could therefore have played a role in the more beneficial health outcomes.

### Sensitization to stressors, episodes, and bouts of substance use

Compared to the Europeans, bipolar patients from the US thus have more stressors, episodes, and substance abuse. Repetition of stressors, episodes of illness, and bouts of substance abuse each result in increased behavioral responsivity or sensitization upon their recurrence. Thus, it is clear that bipolar patients from the US have increased sensitization to stressors, episodes, and substances, and these individually can drive illness progression and deterioration (Post 2016, a, b, c). In addition, each type of sensitization is associated with cross sensitization to the others, yielding an accumulating downward spiral of interacting pathological processes. These three types of sensitization all appear to have an epigenetic basis, manifest by long lasting chemical alterations in one’s DNA, histones (around which DNA is wrapped (Jia et al. 2017)), and micro-RNA. Preventing each type of sensitization thus becomes a major target not only to clinically prevent illness progression, but also limit the associated neurobiological alterations, including accumulation of pathological epigenetic marks on one’s genetic material (Post 2018).

### Treatment studies

As the Network was active in a period when many potential new drugs for bipolar disorder became available on the market, we tested several of these drugs in: (a) randomized placebo-controlled trials; (b) randomized open clinical trials; or (c) proof of principle clinical case series for treatment effectiveness. Whereas large randomized placebo-controlled trials prove efficacy of a drug in an averaged population, mostly with mild to moderate symptoms, they often exclude “real” patients with comorbidities and treatment refractoriness. The unique chance of a non-commercial network such as the SFBN is not only to inform industry about a presumed spectrum of efficacy and tolerability of candidate drugs by pilot studies, but is especially important to inform clinicians about best available treatment options by further characterizing patient subgroups responsive or non-responsive to established medication, even beyond a specific label of a medication. While we conducted few placebo- controlled clinical trials (only assessing the efficacy of omga-3-fatty acids and modafinil in this fashion), this is why our emphasis was on pilot studies and randomized, comparative practical clinical trials as this represents an effective and efficient way of accumulating clinically relevant information about which drugs are most effective and best tolerated.

The results of each of these studies will only be summarized perfunctorily as either showing suggestion or evidence of effectiveness or a lack there of. Details of the methodology and caveats to the interpretation of the results are in each manuscript.

#### Studies in bipolar depression

Daily mood ratings with the LCM by all participants, enabling fine-grained analyses of naturalistic illness course, confirmed that depression is the major burden of BD and thus an unmet need for definition of best interventions (Kupka et al. 2007). Indications of positive effectiveness was seen for: lamotrigine (c) (Suppes et al. 1999); gabapentin (c) (Altshuler et al. 1999); aripiprazole (c) (McElroy et al. 2007b); the monoamine-oxidase inhibitor (MAO-I) tranylcypromine (b) (Nolen et al. 2007); and modafinil (a) in a randomized, placebo-controlled clinical trial (Frye et al. 2007).

In contrast, suggestions for lack of effectiveness were seen for omega -3 fatty acids (a) (Keck et al. 2006); tiagabine (c) (Suppes et al. 2002); and zonisamide (b) (McElroy et al. 2005). The omega -3 fatty acids studies used eight grams of eicosapentaenoic acid (EPA) in two different studies of acute antidepressant efficacy and of longer-term prophylaxis. These represented the largest studies of omega -3 fatty acids to data and yielded the surprising post hoc analysis that while patients younger than age 45 did better on active drug than placebo, older patients over 45 fared more poorly on drug than placebo (Keck et al. 2006). The small pilot study of tiagabine justified our using open uncontrolled methodology as it revealed the new onset of seizures in two patients (Suppes et al. 2002) confirming a previous observation (Grunze et al. 1999). Since open studies are more likely to yield positive findings than double blind controlled studies, our preliminary negative findings for the lack of effectiveness of several drugs are suggestive of a lack of a robust antidepressant response. These open observations could be countered by much larger controlled studies, but our pilot data suggest that these are not ideal candidates to explore further.

Major new findings were seen for the randomized (initially open then blind) administration of three mechanistically different antidepressants (ADs) indicated for unipolar depression, as adjuncts to mood stabilizers in bipolar depression (Leverich et al. 2006; Post et al. 2006, 2003). Here we saw limited acute effectiveness of bupropion, sertraline, and venlafaxine with only about 15% of the intent to treat exposed patients not showing depressive relapses or switches into hypomania or mania following at least 2 months of exposure to an AD. Venlafaxine with it noradrenergic (NE) potency was associated with a higher rate of switching into (hypo)mania compared to bupropion. This replicated earlier data that NE active agents were more likely to induce switches into mania (Sachs et al. 1994; Vieta et al. 2002). These data are important to clinicians making treatment decisions, and validate our decision to do a controlled comparative clinical trial and not a placebo controlled one which would not have readily provided this type of information.

However, of those minority 15% who remained well on an AD for at least two months, we saw that in this instance that continuation of the AD over the next year resulted in fewer depressive relapses than in those whose ADs were discontinued (Altshuler et al. 2009). This AD continuation in this small group of responders was not associated with an increased rate of switching into (hypo)mania.

We also found that bipolar depressed patients with minor manic symptoms (mixed depression) were more like to switch into mania upon adjunctive AD treatment than those with pure depression (Frye et al. 2009) and that those patients who had more previous AD trials (corrected for number of depressive episodes) prior to joining the Network, did more poorly in long term (for 6 months) prospective naturalistic treatment in the Network, suggesting that more prior AD exposure was associated with more treatment refractoriness in general (Post et al. 2012).

#### Studies in mania

Preliminary suggestions of effectiveness were seen in case series for: quetiapine (c) (Suppes et al. 2004) and olanzapine (c) (McElroy et al. 1998) as documented in industry studies and FDA approval. However, the suggestions of the usefulness of gabapentin (c) (Altshuler et al. 1999) and topiramate (c) (McElroy et al. 2000) were not confirmed in industry conducted placebo-controlled clinical trials. An open add-on study using an off–on-off design showed evidence of effectiveness for oxcarbazepine (b) in a small subgroup (Hummel et al. 2002). In this case oxcarbazepine appeared more effective in those with milder symptoms (in contrast to the robust efficacy of carbamazepine in the most severely ill patients).

Mixed results were seen for zonisamide (b)(McElroy et al. 2005) and levetiracetam (c) (Post et al. 2005) where several patients with manic symptoms displayed significant reduction of symptomatology. However, this improvement was countervailed by a fair number of patients who experienced a deterioration of mood.

#### Studies in obesity

In studies intending to demonstrate ability to facilitate weight loss in patients who were obese, suggestions of positive effectiveness on weight were seen with topiramate (b) (McElroy et al. 2007a), sibutramine (b) (McElroy et al. 2007a) and zonisamide (c) (Leverich et al. 2005) although the magnitude of the effect was not very large and some dose limiting side-effects were observed.

## Discussion and conclusion

### Clinical implications for the need for future studies

Some clinicians have taken the position that to label children with a major psychiatric illness such as bipolar disorder would be stigmatizing and that they would be exposed to treatments that had unwanted side effects (Malhi et al. 2020). However, we would instead argue that not properly diagnosing and treating children with bipolar disorder is evidence of stigma toward psychiatry that is not seen in any other branch of medicine (Post et al. 2020b). Moreover, we and others have found that both early onset illness and delay to first treatment are independent risk factors for a poor outcome in adulthood (Post et al. 2010b). Taking a “wait and see” attitude after the manifestation of a mood disorder is fraught with the multiple risks of social and educational dysfunction, affective illness morbidity, acquiring a substance abuse problem, and even suicide.

As noted above we have seen more psychiatric illness in four generations of those from the US compared to Europe, so that it is apparent that the problem is being propelled transgenerationally and needs to be appropriately addressed. An added variable to this pessimistic outlook about the multigenerational transmission of illness are the new data that a cohort effect likely exists (Post et al. 2016c). That is, those who are born in more recent generations are more ill than those who were born in much earlier decades. This cohort effect has been documented in epidemiological based samples (e.g., Bauer et al. 2015; Chengappa et al. 2003) and is evident from our own Network analysis. The more recently born patients (and their parents) are more ill than the earlier born (older) individuals. A cohort effect for the incidence and age of onset of depression (Klerman and Weissman 1989; Lavori et al. 1987) and substance abuse (Stoltenberg et al. 1999; Yamaguchi and Kandel 1984) has been documented for decades, and also appears true for bipolar disorder. Moreover, early age of onset and frequent recurrences of depression do not only affect one’s own course of illness, but also increase the risk of intergenerational transmission of depression to adolescent offspring (Jaffee et al. 2020).

We are aware that societal factors may also play an important role. The availability of socialized healthcare, wage support and disability benefit provisions which are much more generous and easily accessible in Germany and the Netherlands likely play a role in the more beneficial health outcomes. But whatever are the factors driving these cohort effects, they further highlight the multigenerational problems seen in the US. Not only are these problems not self- correcting, but they are if anything getting worse.

We hope this brief overview and incomplete summary of some of the findings in the Network has demonstrated the multiple values and new data gleaned from the study of a longitudinal treatment outcome network with clinical trials imbedded into its naturalistic follow up. The STEP-BD has similarly yielded many insights into the course and treatment of bipolar disorder in adults (Bowden et al. 2012). On the bright side, many new findings have been generated, but on the dark side, the toll taken by the illness remains underestimated and very substantial.

Patients remained ill the majority of the time that they participated in these networks. Poor prognosis factors such as early age of onset, anxiety disorder comorbidity and substance abuse were rampant and associated with an already high rate of suicide attempts (Post et al. 2017a). Medical comorbidities were prominent (Post et al. 2014b) and recent data indicate that patients with bipolar disorder loose a decade or more of life expectancy compared to those in the general community (Lomholt et al. 2019; Nordentoft et al. 2013), propelled mainly by cardiovascular disease which can have an early onset (Goldstein et al. 2015).

While it would be helpful to have another, perhaps larger and more sophisticated, iteration of the Network and STEP-BD, this would appear to be partially doomed by the observations and conclusions that waiting for the illness to enter a full blown or malignant phase of multiple episodes and relative treatment refractoriness might be like closing the barn door once all the animals have escaped. The message of both adult networks and also of the child collaborative COBY study is that we should study the safest interventions that might be preventive (primary preventive for those at ultra-high risk) and which might best head off illness progression once symptoms have appeared (secondary prevention). COBY and multiple other longitudinal studies make clear that childhood onset bipolar disorder is not a benign illness and even those with subthreshold manic symptoms as seen in the BP-NOS subtype are difficult to stabilize and are associated with considerable dysfunction and disability (Birmaher et al. 2009).

We currently know how to preliminarily identify those at high and very high risk by virtue of: 1. Their family history; 2. Adversity in childhood; and 3. Premonitory syndromes and symptoms (Post et al. 2013a, b, 2020b).

Studies among the offspring of parent(s) with bipolar disorder found that over two thirds will develop some disorder, such as an anxiety disorder, depression, a substance use disorder, ADHD or a disruptive behavioral disorder, in addition to the some 20% who will have a bipolar spectrum disorder (Axelson et al. 2015; Duffy et al. 2007; Mesman et al. 2013). If one takes into account the early age of their own bipolar disorder onset in the parents of the offspring, this appears to be an additional risk factor. Using this and other symptoms in the offspring such as anxiety, depression, mood lability, and subthreshold manic symptoms, one can generate a risk calculator of bipolar disorder emergence (Birmaher et al. 2018; Geller et al. 2010). Moreover, the other often premonitory syndromes of anxiety, depression, ADHD, and disruptive behavioral disorders (Faedda et al. 2019) also deserve treatment, and how this is best achieved in child at high risk because of a parent with bipolar disorder has rarely been studied.

For those children who have been diagnosed with a full-blown bipolar disorder, naturalistic data suggest that they have better outcome when treated with lithium than those children who are treated with other agents and mood stabilizers (Geller et al. 2010; Hafeman et al. 2019). A controlled study found that the atypical antipsychotic risperidone was more effective than lithium or valproate, but had more side effects (Geller et al. 2012). There is an urgent need to assess whether other of the many available atypical antipsychotic(s) may be equally or more effective and/or better tolerated. Assessment of the comparative effectiveness and tolerability of a whole host of early intervention studies would be extremely valuable.

Extrapolating from the Network, STEP-BD, and COBY findings, it is readily apparent that what is needed is a longitudinal treatment outcome network for children. This could include children at high risk and those who become or are already ill that builds in practical clinical treatment trials of well-tolerated agents in the youngest children and the later study of more traditional agents in randomized comparative treatment trials in those who are already ill (Post et al. 2020b, 2019).

There are multiple safe agents that are already available that deserve testing for their ability to achieve indicated primary or secondary prevention. These could include omega-3-fatty acids, vitamin D3, L-methyl folate, N-acetylcysteine, acetyl-L-carnitine, minocycline and other anti-inflammatories, and phosphatidylcholine, among many others (Post 2020; Post et al. 2020a, b). While the ultimate trajectory of those a high risk is uncertain, many of the potential interventions themselves appear to have non-specific potential positive effects (such as omega-3-fatty acids potential effectiveness in depression, bipolar disorder, ADHD, and psychosis), so effectiveness could be assessed across a range of syndromes and not just for bipolar disorder (23).

As children at high risk become ill or those who enter a network already ill, a host of comparative clinical trials could be embedded in the network. Many of these could be offered as open randomized clinical trials as most of these have been untested and are at equipoise. We know parents would be willing to have their children entered into clinical trial under these comparative circumstances involving potentially two active agents and not a placebo comparator (Post et al. 2002).

Among children of a bipolar parent who become depressed, it is a sorry state that we have no systematic data on how to best treat that depression. Does one use a traditional antidepressant, lamotrigine, or an atypical such as lurasidone which is FDA approved for treatment of bipolar depression in children aged 10–17 as well as in adults? What are the range of best options for treatment of a first manic episode in children and adolescents; how should they be sequenced; and how should they be used in combinations? What are the best approaches to the multiple comorbidities that accompany childhood onset bipolar disorder? From our Network experience, we highly endorse the conduct of randomized practical trials as the most efficient way to obtain these clinically needed data. While parallel group placebo-controlled trials are the standard required for FDA approval, such clinical trials would be virtually impossible to conduct, expensive, difficult to recruit for, and precluded from industry funding by the lack of patent life of some of the most interesting agents noted above.

When we asked child and adult psychiatrist experts in bipolar disorder how they would treat a child whose mania had improved on risperidone and methylphenidate, but who had residual symptoms of anxiety, ADHD, and oppositional behavior, we found that there was very little agreement about best approaches (Post et al. 2017b). This would not be unexpected, since there are so few studies to inform optimal therapeutics. Merikangas and colleagues (Merikangas et al. 2010) found that 2.2% of adolescents 13–18 years old in the US had a bipolar spectrum disorder, but only 20% were in any kind of treatment. This is unacceptable and should be corrected with a new round of treatment research to guide clinical therapeutics especially in the US where the majority of children with psychiatric disorders are seen in primary care (Anderson et al. 2015).

Compared to the Europeans with bipolar disorder, the US has a unique and greater set of problems across four generations (Post et al. 2015a, 2016a) and now continuing with a new cohort of children with bipolar disorder and related psychiatric conditions in the offspring of parents with bipolar disorder. It is imperative that a longitudinal cohort of children at high risk and those already ill be initiated to begin to address this grave public health crisis. Embedding practical clinical treatment trials into such a network would go a long way to jump starting the field and rapidly provide a modicum of systematic treatment information.

We believe that the methodology and accomplishments outlined here for the Network in adults with bipolar disorder amply demonstrate the potential value of such a longitudinal network for children providing new information about the course and optimal treatment of the early phases of the disorder. Physicians, parents, and children at risk and with dysfunctional symptoms should not be left to suffer from the ongoing deficit of otherwise readily obtainable treatment knowledge.

## Supplementary Information

**Additional file 1.** Supplementary material.

## Data Availability

All data have been previously published in the referenced articles and are in the public domain (Additional file 1).
