# Supplementary material for: 25 Years of the International Bipolar Collaborative Network (BCN)
Source: Int J Bipolar Disord. 2021 Apr 2;9:13. doi: 10.1186/s40345-020-00218-w (PMC8019011; doi:10.1186/s40345-020-00218-w)
Supplement: Supplementary file 1 — Additional file 1. Supplementary material. [file 40345_2020_218_MOESM1_ESM.docx]

## Supplementary material

Publications from the Stanley Foundation Bipolar Network and Bipolar Collaborative Network (updated July 2020)

**Peer- reviewed articles**

1998

McElroy SL, Frye , K. Denicoff, Altshuler L, Nolen W, Kupka R, Suppes T, Keck PE, Leverich GS, Kmetz GF, Post RM. **Olanzapine in treatment-resistant bipolar disorder.** Journal of Affective Disorders 1998; 49: 119-122.

1999

Altshuler LL, Keck Jr PE, McElroy SL, Suppes T, Brown ES, Denicoff K, Frye M, Gitlin M, Hwang S, Goodman R, Leverich G, Nolen W, Kupka R, Post R. **Gabapentin in the acute treatment of refractory bipolar disorder.** Bipolar Disorders 1999; 1: 61-65.

Suppes T, Brown ES, McElroy SL, Keck Jr PE, Nolen W, Kupka R, Frye M, Denicoff KD, Altshuler L, Leverich GS, Post RM. **Lamotrigine for the treatment of bipolar disorder: a clinical case series.** Journal of Affective Disorders 1999; 53: 95-98.

2000

Denicoff KD, Leverich GS, Nolen WA, Rush AJ, McElroy SL, Keck PE, Suppes T, Altshuler LL, Kupka R, Frye MA, Hatef J, Brotman MA, Post RM. **Validation of the prospective NIMH-Life-Chart Method (NIMH-LCM-p) for longitudinal assessment of bipolar illness.** Psychol Med 2000; 30: 1391-1397.

McElroy SL, Suppes T, Keck PE, Frye MA, Denicoff KD, Altshuler LL, Brown ES, Nolen WA, Kupka RW, Rochussen J, Leverich GS, Post RM. **Open-label adjunctive topiramate in the treatment of bipolar disorders.** Biol Psychiatry 2000; 47: 1025-1033.

2001

Post RM, Nolen WA, Kupka RW, Denicoff KD, Leverich GS, Keck PE, McElroy SL, Rush AJ, Suppes T, Altshuler LL, Frye MA, Grunze H, Walden J. **The Stanley Foundation Bipolar Network. 1. Rationale and methods.** Br J Psychiatry 2001; 178 [suppl 41]: s169-s176.

Kupka RW, Nolen WA, Altshuler LL, Denicoff KD, Frye MA, Leverich GS, Keck PE, McElroy SL, Rush AJ, Suppes T, Post RM. **The Stanley Foundation Bipolar Network. 2. Preliminary summary of demographics, course of illness and response to novel treatments.** Br J Psychiatry 2001; 178 [suppl 41]: s177-s183.

Post RM, Altshuler LL, Frye MA, Suppes T, Rush AJ, Keck PE, McElroy SL, Denicoff KD, Leverich GS, Kupka RW, Nolen WA. **Rate of switch in bipolar patients prospectively treated with second-generation antidepressants as augmentation to mood stabilizers.** Bipolar Disorders 2001; 3: 259-265.

McElroy SL, Altshuler LL, Suppes T, Keck PE, Frye MA, Denicoff KD, Nolen WA, Kupka RW, Leverich GS, Rochussen JR, Rush AJ, Post RM. **Axis I psychiatric comorbidity and its relationship to historical illness variables in 288 patients with bipolar disorder.** Am J Psychiatry 2001; 158: 420-426.

Leverich GS, Nolen WA, Rush AJ, McElroy SL, Keck PE, Denicoff KD, Suppes T, Altshuler LL, Kupka R, Kramlinger KG, Post RM. **The Stanley Foundation Bipolar Treatment Outcome Network. I. Longitudinal methodology.** Journal of Affective Disorders 2001; 67: 33-44.

Suppes T, Leverich GS, Keck PE, Nolen WA, Denicoff KD, Altshuler LL, McElroy SL, Rush AJ, Kupka R, Frye MA, Bickel M, Post RM. **The Stanley Foundation Bipolar Treatment Outcome Network. II. Demographics and illness characteristics of the first 261 patients.** Journal of Affective Disorders 2001; 67: 45-49.

2002

Leverich GS, McElroy SL, Suppes T, Keck PE, Denicoff KD, Nolen WA, Altshuler LL, Rush AJ, Kupka R, Frye MA, Autio KA, Post RM. **Early Physical and Sexual Abuse Associated with an Adverse Course of Bipolar Illness.** Biological Psychiatry 2002; 51: 288-297.

McElroy SL, Frye MA, Suppes T, Dhavale D, Keck PE, Leverich GS, Altshuler L, Denicoff KD, Nolen WA, Kupka R, Grunze H, Walden J, Post RM. **Correlates of overweight and obesity in 644 patients with bipolar disorder.** J Clin psychiatry 2002; 63: 207-213.

Suppes T, Chisholm KA, Dhavale D, Frye MA, Altshuler LL, McElroy SL, Keck PE, Nolen WA, Kupka R, Denicoff KD, Leverich GS, Rush AJ, Post RM. **Tiagabine in treatment refractory bipolar disorder: a clinical case series.** Bipolar Disorders 2002; 5; 283-289.

Post RM, Leverich GS, Denicoff KD, Altshuler LL, Frye MA, Rush J, Suppes T, Kramlinger K, Keck PEJr, McElroy SL, Nolen WA, Kupka R, Walden J, Grunze H. The Stanley Foundation Bipolar Network. Pt 1: New findings on suicide attempts, substance abuse, obesity, and more. *Current Psychiatry* 2002; 1: 26-32.

Post RM, Leverich GS, Denicoff KD, Altshuler LL, Frye MA, Rush J, Suppes T, Kramlinger K, Keck PEJr, McElroy SL, Nolen WA, Kupka R, Walden J, Grunze H. **The Stanley Foundation Bipolar Network. Pt. 2: Efficacy of adjunctive therapies for treatment-resistant patients**. *Current Psychiatry* 2002; 1: 36-44.

Kupka RW, Nolen WA, Post RM, McElroy SL, Altshuler LL, Denicoff KD, Frye MA, Keck Jr PE, Leverich GS, Rush AJ, Suppes T, Pollio C, Drexhage HA. **High Rate of Autoimmune Thyroiditis in Bipolar Disorder: Lack of Association with Lithium Exposure.** Biological Psychiatry 2002; 51: 305-311.

2003

Breunis MN, Kupka RW, Nolen WA, Suppes T, Denicoff KD, Leverich GS, Post RM, Drexhage HA. **High numbers of circulating activated T cells and raised levels of serum IL-2 receptor in bipolar disorder.** Biological Psychiatry 2003; 53:157-165.

Leverich GS, Altshuler LL, Frye MA, Suppes T, Keck PE, McElroy SL, Denicoff KD, Obrocea G, Nolen WA, Kupka R, Walden J, Grunze H, Perez S, Luckenbaugh DA, Post RM. **Factors Associated With Suicide Attempts in 648 Patients With Bipolar Disorder in the Stanley Foundation Bipolar Network.** Journal of Clinical Psychiatry 2003; 64:506-515.

Post RM, Denicoff KD, Leverich GS, Altshuler LL, Frye MA, Suppes TM, Rush AJ, Keck PE, McElroy SL, Luckenbaugh DA, Pollio C, Kupka R, Nolen WA. **Morbidity in 258 Bipolar Outpatients Followed for 1 Year With Daily Prospective Ratings on the NIMH Life Chart Method.** Journal of Clinical Psychiatry. 2003; 64:680-690.

Frye MA, Altshuler LL, McElroy SL, Suppes T, Keck PE, Denicoff K, Nolen WA,

Kupka R, Leverich GS, Pollio C, Grunze H, Walden J, Post RM. **Gender differences in prevalence, risk, and clinical correlates of alcoholism comorbidity in bipolar disorder.** American Journal of Psychiatry 2003; 160:883-889.

Altshuler L, Suppes T, Black D, Nolen WA, Keck PE Jr, Frye MA, McElroy S, Kupka R, Grunze H, Walden J, Leverich G, Denicoff K, Luckenbaugh D, Post R. **Impact of antidepressant discontinuation after acute bipolar depression remission on rates of depressive relapse at 1-year follow-up.** American Journal of Psychiatry. 2003; 160:1252-1262.

Keck PE, Jr, McElroy SL, Rochussen-Havens J, Altshuler LL, Nolen WA, Frye MA, Suppes T, Denicoff KD, Kupka R, Leverich GS, Rush AJ, Post RM. **Psychosis in bipolar disorder: phenomenology and impact on morbidity and course of illness.** Comprehensive Psychiatry 2003; 4:263-269.

Suppes T, Chisholm KA, Dhavale D, Frye MA, Altshuler LL, McElroy SL, Keck PE, Nolen WA, Kupka R, Denicoff KD, Leverich GS, Rush AJ, Post RM. **Tiagabine en el tratamiento del trastorno bipolar refractorio: una serie de casos clinicos.** Bipolar Disorders (Ed Esp) 2003; 1; 135-141.

Kupka RW, Luckenbaugh DA, Post RM, Leverich GS, Nolen WA. **Rapid and non-rapid cycling bipolar disorder: a meta-analysis of clinical studies.** Journal of Clinical Psychiatry 2003; 64: 1483-1494.

Post RM, Leverich GS, Altshuler LL, Frye MA, Suppes TM, Keck PE Jr, McElroy SL, Kupka R, Nolen WA, Grunze H, Walden J. **An overview of recent findings of the Stanley Foundation Bipolar Network (Part I).** Bipolar Disorders 2003; 5:310-9.

Post RM, Leverich GS, Nolen WA, Kupka RW, Altshuler LL, Frye MA, Suppes T,

McElroy S, Keck P, Grunze H, Walden J. **A re-evaluation of the role of antidepressants in the treatment of bipolar depression: data from the Stanley Foundation Bipolar Network.** Bipolar Disorders 2003; 5:396-406.

2004

Nolen WA, Luckenbaugh DA, Altshuler LL, Suppes T, McElroy SL, Frye MA, Kupka RW, Keck PE Jr, Leverich GS, Post RM. **Correlates of 1-year prospective outcome in bipolar disorder: results from the stanley foundation bipolar network.** American Journal of Psychiatry 2004; 161:1447-54.

Suppes T, McElroy SL, Keck PE, Altshuler L, Frye MA, Grunze H, Leverich GS, Nolen WA, Chisholm K, Dennehy EB, Post RM. **Use of quetiapine in bipolar disorder: a case series with prospective evaluation**. Int Clin Psychopharmacol. 2004; 19(3):173-4

2005

Post RM, Altshuler LL, Frye MA, Suppes T, Keck Jr PE, McElroy SL, Leverich GS, Kupka RW, Nolen WA, Walden J, Grunze H. **An overview of the findings of the Stanley Foundation Bipolar Network (SFBN) and the Bipolar Collaborative Network (BCN): a focus on anticonvulsants.** Aspects of Affect 2005; 1: 8-17.

Leverich GS, McElroy SL, Altshuler LL, Frye MA, Grunze H, Keck Jr PE, Kupka RW, Nolen WA, Suppes T, Walden J, Post RM. **The anticonvulsant zonisamide in bipolar illness: clinical response and weight loss.** Aspects of Affect 2005; 1: 53-56.

Rasgon NL, Altshuler LL, Fairbanks L, Elman S, Bitran J, Labarca R, Saad M, Kupka R, Nolen WA, Frye MA, Suppes T, McElroy SL, Keck PE Jr, Leverich G, Grunze H, Walden J, Post R, Mintz J. **Reproductive function and risk for PCOS in women treated for bipolar disorder.** Bipolar Disorders 2005;7:246-259.

McElroy SL, Suppes T, Keck PE Jr, Black D, Frye MA, Altshuler LL, Nolen WA, Kupka RW, Leverich GS, Walden J, Grunze H, Post RM. **Open-label adjunctive zonisamide in the treatment of bipolar disorders: a prospective trial.** J Clin Psychiatry. 2005;66:617-624.

Kupka RW, Luckenbaugh DA, Post RM, Suppes T, Altshuler LL, Keck PE Jr, Frye MA, Denicoff KD, Grunze H, Leverich GS, McElroy SL, Walden J, Nolen WA. **Comparison of rapid-cycling and non-rapid-cycling bipolar disorder based on prospective mood ratings in 539 outpatients.** Am J Psychiatry 2005; 162: 1273-1280.

Post RM, Altshuler LL, Frye MA, Suppes T, McElroy SL, Keck PE Jr, Leverich GS, Kupka R, Nolen WA, Luckenbaugh DA, Walden J, Grunze H. **Preliminary observations on the effectiveness of**

**levetiracetam in the open adjunctive treatment of refractory bipolar disorder.** J Clin Psychiatry. 2005;66:370-374.

Suppes T, Mintz J, McElroy SL, Altshuler LL, Kupka RW, Frye MA, Keck PE Jr, Nolen WA, Leverich GS, Grunze H, Rush AJ, Post RM. **Mixed hypomania in 908 patients with bipolar disorder evaluated prospectively in the Stanley Foundation Bipolar Treatment Network: a sex-specific phenomenon.** Archives of General Psychiatry 2005;62:1089-1096.

2006

Altshuler LL, Suppes T, Black DO, Nolen WA, Leverich G, Keck PE Jr, Frye MA, Kupka R, McElroy SL, Grunze H, Kitchen CM, Post R. **Lower Switch Rate in Depressed Patients With Bipolar II Than Bipolar I Disorder Treated Adjunctively With Second-Generation Antidepressants.** Am J Psychiatry 2006;163:313-315.

Leverich GS, Altshuler LL, Frye MA, Suppes T, McElroy SL, Keck PE Jr, Kupka RW, Denicoff KD, Nolen WA, Grunze H, Martinez MI, Post RM. **Risk of switch in mood polarity to hypomania or mania in patients with bipolar depression during acute and continuation trials of venlafaxine, sertraline, and bupropion as adjuncts to mood stabilizers.** Am J Psychiatry 2006;163:232-239.

Keck PE Jr, Mintz J, McElroy SL, Freeman MP, Suppes T, Frye MA, Altshuler LL, Kupka R, Nolen WA, Leverich GS, Denicoff KD, Grunze H, Duan N, Post RM. **Double-Blind, Randomized, Placebo-Controlled Trials of Ethyl-Eicosapentanoate in the Treatment of Bipolar Depression and Rapid Cycling Bipolar Disorder.** Biol Psychiatry. 2006 Jun 27; [Epub ahead of print]

Post RM, Altshuler LL, Leverich GS, Frye MA, Nolen WA, Kupka RW, Suppes T, McElroy S, Keck PE, Denicoff KD, Grunze H, Walden J, Kitchen CM, Mintz J. **Mood switch in bipolar depression: comparison of adjunctive venlafaxine, bupropion and sertraline.** Br J Psychiatry 2006;189:124-31.

Altshuler LL, Post RM, Black DO, Keck PE Jr, Nolen WA, Frye MA, Suppes T, Grunze H, Kupka RW, Leverich GS, McElroy SL, Walden J, Mintz J. **Subsyndromal depressive symptoms are associated with functional impairment in patients with bipolar disorder: results of a large, multisite study.** J Clin Psychiatry 2006;67:1551-1560.

Post RM, Altshuler LL, Frye MA, Suppes T, McElroy S, Keck PE Jr, Leverich GS, Kupka R, Nolen WA, Grunze H. **New findings from the Bipolar Collaborative Network: clinical implications for therapeutics.** Curr Psychiatry Rep. 2006; 8: 489-497.

2007

Levander E, Frye MA, McElroy S, Suppes T, Grunze H, Nolen WA, Kupka R, Keck PE Jr, Leverich GS, Altshuler LL, Hwang S, Mintz J, Post RM. **Alcoholism and anxiety in bipolar illness: Differential lifetime anxiety comorbidity in bipolar I women with and without alcoholism.** J Affect Disord. 2007; 101: 211-7.

Leverich GS, Post RM, Keck PE, Altshuler LL, Frye MA, Kupka RW, Nolen WA, Suppes T, McElroy SL, Grunze H, Denicoff K, Moravec MK, Luckenbaugh D. **The poor prognosis of childhood-onset bipolar disorder.** J Pediatr. 2007;150:485-490.

Nolen WA, Kupka RW, Hellemann G, Frye MA, Altshuler LL, Leverich GS, Suppes T, Keck PE, McElroy S, Grunze H, Mintz J, Post RM. **Tranylcypromine vs. lamotrigine in the treatment of refractory bipolar depression: a failed but clinically useful study**. Acta Psychiatr Scand. 2007;115:360-365.

Kupka RW, Altshuler LL, Nolen WA, Suppes T, Luckenbaugh DA, Leverich GS, Frye MA, Keck PE Jr., McElroy SL, Grunze H, Post RM. **Three times more depression than mania in both bipolar I and bipolar II disorder.** Bipolar Disorders 2007; 9: 531-535.

McElroy SL, Frye MA, Altshuler LL, Suppes T, Hellemann G, Black D, Mintz J, Kupka R, Nolen W, Leverich GS, Denicoff KD, Post RM, Keck PE. **A 24-week, randomized, controlled trial of adjunctive sibutramine versus topiramate in the treatment of weight gain in overweight or obese patients with bipolar disorders.** Bipolar Disord. 2007;9:426-434.

Suppes T, Kelly DI, Keck PE Jr, McElroy SL, Altshuler LL, Mintz J, Frye MA, Nolen WA, Luckenbaugh DA, Post RM, Leverich GS, Kupka RW, Grunze H. **Quetiapine for the continuation treatment of bipolar depression: naturalistic prospective case series from the Stanley Bipolar Treatment Network**. Int Clin Psychopharmacol 2007;22:376-381.

2008

Post RM, Luckenbaugh DA, Leverich GS, Altshuler LL, Frye MA, Suppes T, Keck PE, McElroy SL, Nolen WA, Kupka R, Grunze H, Walden J. **Incidence of childhood-onset bipolar illness in the USA and Europe.** Br J Psychiatry. 2008;192:150-151.

Shivakumar G, Bernstein IH, Suppes T; Stanley Foundation Bipolar Network, Keck PE, McElroy SL, Altshuler LL, Frye MA, Nolen WA, Kupka RW, Grunze H, Leverich GS, Mintz J, Post RM. **Are bipolar mood symptoms affected by the phase of the menstrual cycle?** J Womens Health 2008;17:473-478.

2009

Frye MA, Helleman G, McElroy SL, Altshuler LL, Black DO, Keck PE Jr, Nolen WA, Kupka R, Leverich GS, Grunze H, Mintz J, Post RM, Suppes T. **Correlates of treatment-emergent mania associated with antidepressant treatment in bipolar depression.** Am J Psychiatry. 2009;166:164-172.

Altshuler LL, Post RM, Hellemann G, Leverich GS, Nolen WA, Frye MA, Keck PE Jr, Kupka RW, Grunze H, McElroy SL, Sugar CA, Suppes T. **Impact of antidepressant continuation after acute positive or partial treatment response for bipolar depression: a blinded, randomized study.** J Clin Psychiatry 2009;70:450-457.

2010

Altshuler LL, Kupka RW, Hellemann G, Frye MA, Sugar CA, McElroy SL, Nolen WA, Grunze H, Leverich GS, Keck PE Jr, Zermeno M, Post RM, Suppes T**. Gender and Depressive Symptoms in 711 Patients with Bipolar Disorder Evaluated Prospectively in the Stanley Foundation Bipolar Treatment Outcome Network.** Am J Psychiatry. 2010;167:708-715.

Post RM, Leverich GS, Kupka RW, Keck PE, McElroy SL, Altshuler LL, Frye MA, Luckenbaugh DA, Rowe M, Grunze H, Suppes T, Nolen WA. **Early-onset bipolar disorder and treatment delay are risk factors for poor outcome in adulthood.** J Clin Psychiatry 2010;**71**:864-872

Post RM, Altshuler LL, Frye MA, Suppes T, Keck PE Jr, McElroy SL, Leverich GS,

Luckenbaugh DA, Rowe M, Pizzarello S, Kupka RW, Grunze H, Nolen WA**. Complexity of pharmacologic treatment required for sustained improvement in outpatients with bipolar disorder**. J Clin Psychiatry. 2010;71:1176-1186.

2011

Post RM, Leverich GS, Altshuler LL, Frye MA, Suppes T, Keck PE, McElroy SL, Nolen

WA, Kupka R, Grunze H, Walden J, Rowe M. **Differential clinical characteristics, medication usage, and treatment response of bipolar disorder in the US versus The Netherlands and Germany.** Int Clin Psychopharmacol. 2011;26:96-106.

McElroy SL, Frye MA, Hellemann G, Altshuler L, Leverich GS, Suppes T, Keck PE,

Nolen WA, Kupka R, Post RM. **Prevalence and correlates of eating disorders in 875 patients with bipolar disorder.** J Affect Disord. 2011;128:191-198.

2012

Post RM, Leverich GS, Altshuler LL, Frye MA, Suppes T, McElroy SL, Keck PE Jr, Nolen WA, Rowe M, Kupka RW, Grunze H, Goodwin FK. **Relationship of prior antidepressant exposure to long-term prospective outcome in bipolar I disorder outpatients.** J Clin Psychiatry. 2012;73:924-930

2013

Post RM, Altshuler LL, Leverich GS, Frye MA, Suppes T, McElroy SL, Keck PE Jr, Nolen WA, Kupka RW, Grunze H, Rowe M. **Role of childhood adversity in the development of medical co-morbidities associated with bipolar disorder.** J Affect Disord. 2013;147:288-294.

Post RM, Altshuler L, Leverich G, Nolen W, Kupka R, Grunze H, Frye M, Suppes T,

McElroy S, Keck P, Rowe M. **More stressors prior to and during the course of bipolar illness in patients from the United States compared with the Netherlands and Germany.** Psychiatry Res. 2013 Sep 7. pii: S0165-1781(13)00467-8. doi: 10.1016/j.psychres.2013.08.007. [Epub ahead of print]

Post RM, Leverich GS, Kupka R, Keck P Jr, McElroy S, Altshuler L, Frye MA, Luckenbaugh DA, Rowe M, Grunze H, Suppes T, Nolen WA**. Increased parental history of bipolar disorder in the United States: association with early age of onset**. Acta Psychiatr Scand. 2013 Oct 19. doi: 10.1111/acps.12208. [Epub ahead of print]

2014

Post RM, Altshuler LL, Leverich GS, Nolen WA, Kupka R, Grunze H, Frye MA, Suppes T, McElroy SL, Keck PE Jr, Rowe M. **Illness progression as a function of independent and accumulating poor prognosis factors in outpatients with bipolar disorder in the United States**. Prim Care Companion CNS Disord. 2014;16(6).

Post RM, Altshuler LL, Leverich GS, Frye MA, Suppes T, McElroy SL, Keck PE Jr, Nolen WA, Kupka RW, Grunze H, Rowe M. **More medical comorbidities in patients with bipolar disorder from the United States than from the Netherlands and Germany.** J Nerv Ment Dis. 2014;202:265-270.

Post RM, Altshuler L, Kupka R, McElroy S, Frye MA, Rowe M, Leverich GS, Grunze H, Suppes T, Keck PE Jr, Nolen WA. **More pernicious course of bipolar disorder in the United States than in many European countries: Implications for policy and treatment.** J Affect Disord. 2014;160:27-33.

2015

Post RM, Altshuler L, Kupka R, McElroy SL, Frye MA, Rowe M, Grunze H, Suppes T, Keck PE Jr, Leverich GS, Nolen WA. **Multigenerational Positive Family History of Psychiatric Disorders Is Associated With a Poor Prognosis in Bipolar Disorder.** J Neuropsychiatry Clin Neurosci. 2015;27:304-310.

Post RM, Altshuler LL, Kupka R, McElroy SL, Frye MA, Rowe M, Leverich GS, Grunze H, Suppes T, Keck PE Jr, Nolen WA. **Verbal abuse, like physical and sexual abuse, in childhood is associated with an earlier onset and more difficult course of bipolar disorder.** Bipolar Disord. 2015;17:323-330..

Post RM, Altshuler L, Leverich GS, Frye MA, Suppes T, McElroy SL, Keck PE Jr, Nolen WA, Kupka RW, Grunze H, Rowe M. **Relationship of clinical course of illness variables to medical comorbidities in 900 adult outpatients with bipolar disorder**. Compr Psychiatry. 2015; 56: 21-28

Post RM, Leverich GS, Kupka R, Keck PE Jr, McElroy SL, Altshuler LL, Frye MA, Rowe M, Grunze H, Suppes T, Nolen WA. **Increases in multiple psychiatric disorders in parents and grandparents of patients with bipolar disorder from the USA compared with The Netherlands and Germany.** Psychiatr Genet. 2015; 5:194-200.

2016

Post RM, Altshuler LL, Kupka R, McElroy SL, Frye MA, Rowe M, Grunze H, Suppes T, Keck PE, Leverich GS, Nolen WA). **More illness in offspring of bipolar patients from the U.S. compared to Europe.** J Affect Disord. 2016;191:180-6..

Post RM, Leverich GS, Kupka R, Keck PE Jr, McElroy SL, Altshuler LL, Frye MA, Rowe M, Grunze H, Suppes T, Nolen WA. **Clinical correlates of sustained response to individual drugs used in naturalistic treatment of patients with bipolar disorder.** Compr Psychiatry. 2016;66:146-156

Miller S, Suppes T, Mintz J, Hellemann G, Frye MA, McElroy SL, Nolen WA, Kupka R, Leverich GS, Grunze H, Altshuler LL, Keck PE, Post RM. **Mixed Depression in Bipolar Disorder: Prevalence Rate and Clinical Correlates During Naturalistic Follow-Up in the Stanley Bipolar Network**. Am J Psychiatry. 2016 Apr 15:appiajp201615091119. [Epub ahead of print]

Post RM, Altshuler LL, Kupka R, McElroy SL, Frye MA, Rowe M, Grunze H, Suppes T, Keck PE Jr, Leverich GS, Nolen WA. **Age of onset of bipolar disorder: Combined effect of childhood adversity and familial loading of psychiatric disorders.** J Psychiatr Res. 2016 Oct;81:63-70. doi: 10.1016/j.jpsychires.2016.06.008. Epub 2016 Jun 16.

Post RM, Altshuler LL, Kupka R, McElroy SL, Frye MA, Rowe M, Grunze H, Suppes T, Keck PE Jr, Leverich GS, Nolen WA. **Age at Onset of Bipolar Disorder Related to Parental and Grandparental Illness Burden.** J Clin Psychiatry. 2016 Oct;77(10):e1309-e1315.

Post RM, Kupka R, Keck PE Jr, McElroy SL, Altshuler LL, Frye MA, Rowe M, Grunze H, Suppes T, Leverich GS, Nolen WA. **Further Evidence of a Cohort Effect in Bipolar Disorder: More Early Onsets and Family History of Psychiatric Illness in More Recent Epochs.** J Clin Psychiatry. 2016 Aug;77(8):1043-9. doi: 10.4088/JCP.15m10121.

2017

Post RM, Altshuler LL, Kupka R, McElroy SL, Frye MA, Rowe M, Grunze H, Suppes T, Keck PE Jr, Nolen WA. **Illnesses in siblings of US patients with bipolar disorder relate to multigenerational family history and patients severity of illness.** J Affect Disord. 2017;207:313-319.

Post RM, Altshuler LL, Kupka R, McElroy SL, Frye MA, Rowe M, Grunze H, Suppes T, Keck PE Jr, Leverich GS, Nolen WA. **More childhood onset bipolar disorder in the United States than Canada or Europe: Implications for treatment and prevention.** Neurosci Biobehav Rev. 2017;74 (Pt A):204-213.

Riemann G, Weisscher N, Post RM, Altshuler L, McElroy S, Frye MA, Keck PE Jr, Leverich GS, Suppes T, Grunze H, Nolen WA, Kupka RW. **The relationship between self-reported borderline personality features and prospective illness course in bipolar disorder.** Int J Bipolar Disord. 2017;5:31.

2018

Post RM, Leverich GS, McElroy S, Kupka R, Suppes T, Altshuler L, Nolen W, Frye M, Keck P, Grunze H, Hellemann G. **Prevalence of axis II comorbidities in bipolar disorder: relationship to mood state.** Bipolar Disord. 2018;20:303-312

Post RM, McElroy S, Kupka R, Suppes T, Hellemann G, Nolen W, Frye M, Keck P, Grunze H, Rowe M. **Axis II Personality Disorders Are Linked to an Adverse Course of Bipolar Disorder.** J Nerv Ment Dis. 2018;206:469-472.

Post RM, Altshuler LL, Kupka R, McElroy SL, Frye MA, Rowe M, Grunze H, Suppes T, Keck PE Jr, Leverich GS, Nolen WA. **Multigenerational transmission of liability to psychiatric illness in offspring of parents with bipolar disorder.** Bipolar Disord. 2018 Jun 21.

Post RM, Altshuler LL, Kupka R, McElroy SL, Frye MA, Rowe M, Grunze H, Suppes T, Keck PE Jr, Nolen WA. **More assortative mating in US compared to European parents and spouses of**

**patients with bipolar disorder: implications for psychiatric illness in the**

**offspring.** Eur Arch Psychiatry Clin Neurosci. 2018 Aug 11.

2019

Altshuler LL, Suppes T, Black DO, Nolen WA, Leverich G, Keck PE Jr, Frye MA, Kupka R, McElroy SL, Grunze H, Kitchen CMR, Post R. **Lower Switch Rate in Depressed Patients With Bipolar II Than Bipolar I Disorder Treated Adjunctively With Second-Generation Antidepressants.** Focus (Am Psychiatr Publ). 2019;17:322-324.

Fredskild MU, Mintz J, Frye MA, McElroy SL, Nolen WA, Kupka R, Grunze H, Keck PE Jr, Post RM, Kessing LV, Suppes T. **Adding Increased Energy or Activity to Criterion (A) of the DSM-5 Definition of Hypomania and Mania: Effect on the Diagnoses of 907 Patients From the Bipolar Collaborative Network.** J Clin Psychiatry. 2019 Oct 29;80(6). pii: 19m12834. doi: 10.4088/JCP.19m12834.

Bennett F, Hodgetts S, Close A, Frye M, Grunze H, Keck P, Kupka R, McElroy S, Nolen W, Post R, Schärer L, Suppes T, Sharma AN. **Predictors of psychosocial outcome of bipolar disorder: data from the Stanley Foundation Bipolar Network.** Int J Bipolar Disord. 2019 Dec 16;7(1):28. doi: 10.1186/s40345-019-0169-5.

van der Markt A, Klumpers UM, Draisma S, Dols A, Nolen WA, Post RM, Altshuler LL, Frye MA, Grunze H, Keck PE Jr, McElroy SL, Suppes T, Beekman AT, Kupka RW. **Testing a clinical staging model for bipolar disorder using longitudinal life chart data.** Bipolar Disord. 2019;21:228-234.

2020

Post RM, Leverich GS, McElroy S, Kupka R, Suppes T, Altshuler L, Nolen W, Frye M, Keck P, Grunze H, Rowe M. **Relationship of comorbid personality disorders to prospective outcome in bipolar disorder**. J Affect Disord. 2020 Jul 16;276:147-151. doi: 10.1016/j.jad.2020.07.031. Epub ahead of print.

Post RM, Altshuler LL, Kupka R, McElroy SL, Frye MA, Rowe M, Grunze H, Suppes T, Keck PE Jr, Nolen WA. **Double jeopardy in the United States: Early onset bipolar disorder and treatment delay**. Psychiatry Res. 2020 Jul 3;292:113274. doi: 10.1016/j.psychres.2020.113274. Online ahead of print.
